# Co-expression of Nisin Z and Leucocin C as a Basis for Effective Protection Against *Listeria monocytogenes* in Pasteurized Milk

**DOI:** 10.3389/fmicb.2018.00547

**Published:** 2018-03-23

**Authors:** Yuxin Fu, Dongdong Mu, Wanjin Qiao, Duolong Zhu, Xiangxiang Wang, Fulu Liu, Haijin Xu, Per Saris, Oscar P. Kuipers, Mingqiang Qiao

**Affiliations:** ^1^Key Laboratory of Molecular Microbiology and Technology, Ministry of Education, Nankai University, Tianjin, China; ^2^Key Laboratory for Agricultural Products Processing of Anhui Province, Hefei University of Technology, Hefei, China; ^3^Department of Food and Environmental Sciences, University of Helsinki, Helsinki, Finland; ^4^Department of Molecular Genetics, Groningen Biomolecular Sciences and Biotechnology Institute, University of Groningen, Groningen, Netherlands

**Keywords:** foodborne pathogens, *Listeria monocytogenes*, nisin, leucocin C, *Lactococcus lactis*, antimicrobial activity

## Abstract

Nisin, an important bacteriocin from *Lactococcus lactis* subsp., is primarily active against various Gram-positive bacteria. Leucocin C, produced by *Leuconostoc carnosum* 4010, is a class IIa bacteriocin used to inhibit the growth of *Listeria monocytogenes.* Because two bacteriocins have different modes of action, the combined use of them could be a potential strategy for effective inhibition of foodborne pathogens. In this study, *L. lactis* N8-r-lecCI (N8 harboring *lecCI* gene) coexpressing nisin–leucocin C was constructed based on the food-grade carrier *L. lactis* N8. Production of both bacteriocins was stably maintained. Antimicrobial measurements showed that the recombinant strain is effectively against *Listeria monocytogenes* and *Staphylococcus aureus* and moderately against *Salmonella enterica* serovar Enteritidis and *Escherichia coli* because of its stronger antibacterial activity than the parental strain, this result first demonstrated that the co-expression of nisin and leucocin C results in highly efficient antimicrobial activity. The checkerboard assay showed that the antibacterial activity of *L. lactis* N8-r-lecCI supernatant was enhanced in the presence of low concentration of EDTA. Analysis of the scanning electron microscope image showed the biggest cellular morphology change in *L. monocytogenes* treated with a mixture of EDTA and *L. lactis* N8-r-lecCI supernatant. The practical effect was verified in pasteurized milk through time-kill assay. The *L. lactis* N8-r-lecCI strain expressing both nisin and leucocin C has a promising application prospect in pasteurized milk processing and preservation because of its strong antibacterial activity.

## Introduction

Pathogen control is a significant issue in the food industry. Foodborne diseases can spread further and more rapidly than before because of the increasing transnational trade, travel, and migration (Kaferstein et al., 1997). Although various measures have been implemented to decrease infections caused by foodborne pathogens (Gandhi and Chikindas, 2007), foodborne disease outbreaks from microbial contamination, chemicals, and toxins remain prevalent worldwide (Velusamy et al., 2010). For example, in the outbreak of listeriosis in the United States in September 2011, a total of 146 invasive illnesses, 30 deaths, and 1 miscarriage were recorded (Laksanalamai et al., 2012). The foodborne pathogen Listeria, the causative agent of listeriosis (Yousef and Lou, 1999), can grow at refrigeration temperatures and adapt to low pH and high salt concentration (Cole et al., 1990). As such, this pathogen can overcome various bacteriostatic measures, increasing the difficulty of anti-*listeria* treatments.

Researchers have developed various methods for controlling foodborne pathogens to reduce their potential risks to human health (Gandhi and Chikindas, 2007). Such methods include the addition of bacteriocins and bacteriocin-producing lactic acid bacteria (LAB) in food (Roberts and Zottola, 1993) as effective alternatives to chemical preservatives (Lucera et al., 2012). *Lactococcus*, *Lactobacillus*, *Leuconostoc*, and other LAB can secrete bacteriocins (Barefoot and Klaenhammer, 1983; Klaenhammer, 1993; Wan et al., 2013, 2015), which are ribosomally synthesized and extracellularly released peptides with antimicrobial activity. LAB bacteriocin has been widely characterized because of their “food-grade quality” and industrial importance (Barbosa et al., 2017). Nisin, an important bacteriocin from LAB, is primarily active against Gram-positive bacteria (Delves-Brougthon, 1990). Nisin is used as a food protection agent in more than 60 countries and regions worldwide because of its low toxicity and high efficiency. In addition to nisin, one of the available commercial antibacterial agents (Barbosa et al., 2017), other bacteriocins exhibit promising perspectives. Class IIa bacteriocin piscicolin P126 displays antilisterial activity in milk and Camembert cheese (Wan et al., 1997). Enterocin AS-48 is active against *Alicyclobacillus acidoterrestris*, which is a spoilage-causing bacterium in fruit juices (Grande et al., 2005). Leucocin C kills the foodborne pathogen *Listeria monocytogenes*, and leucocin C-producing strain *Leuconostoc carnosum* 4010 is used as protective culture in meat products (Wan et al., 2013).

Bacteriocins confer protection against foodborne pathogens; however, single application of bacteriocins is hindered by their limited activity spectrum, high concentration demand and the prevalence of antimicrobial resistance. Several studies have demonstrated that some bacteriocins show additive or synergistic effects when used in combination with other antimicrobial agents; in this regard, combining bacteriocins is a good alternative to the use of single bacteriocin for effective pathogen inhibition. For example, the combination of nisin and its variants with carvacrol and/or citric acid inhibited Gram-negative foodborne pathogens (Campion et al., 2017). The activity of lysozyme and nisin against *L. monocytogenes* and *Escherichia coli* was enhanced with the addition of EDTA (Branen and Davidson, 2004). The efficacy of the antibiotics polymyxin and colistin for preventing the biofilm formation of *Pseudomonas aeruginosa* increased when they were combined with nisin treatment (Field et al., 2016).

In this study, a strain of *L. lactis* stably coexpressing nisin–leucocin C was constructed to control a wide range of foodborne pathogens, including *L. monocytogenes*. LAB are generally recognized as safe (GRAS) microorganisms and widely used in dairy and fermented food (Roberts and Zottola, 1993; O’Sullivan et al., 2002; Swetwiwathana and Visessanguan, 2015). Application of bacteriocin-producing LAB strains may be more effective than direct addition of preservatives because of the continuous *in situ* production of bacteriocins (Langa et al., 2017). However, the use of genetically modified lactic acid bacteria (GM-LAB) was not widely applied and accepted in many Countries (Sybesma et al., 2006). Risk assessment and expected benefits will determine the future use of GM-LAB in the domains of food technology and health (Renault, 2002; LeBlanc et al., 2010; Capozzi et al., 2012). We aimed to construct a *L. lactis* strain, based on food-grade biotechnological strategy, with improved antimicrobial activity and broad antimicrobial spectrum without using genes from non-GRAS bacteria and antibiotics selection pressure for maintaining genetic stability.

## Materials and Methods

### Plasmids, Strains, and Growth Conditions

*Lactococcus lactis* N8 (N8) (Qiao et al., 1996), a wild-type (WT) nisin Z producer, and *L. lactis* NZ9000 (NZ9000) (Kuipers et al., 1998), which is usually used as cloning host, were cultured without agitation at 30°C in SGM17 broth [M17 broth supplemented with 0.5% (w/v) glucose and 0.55% (w/v) sucrose]. When needed the broth was supplemented with 10 ng/mL nisin for induction expression. Antibiotic susceptibility screening was conducted using 150 μg/mL erythromycin (Em) and 15 μg/mL chloramphenicol (Cm) for *E. coli* DH5α and 5 μg/mL Em and 5 μg/mL Cm for *L. lactis*.

*Escherichia coli* O157:H7, *L. monocytogenes* WSLC 1018, and *Micrococcus luteus* A1CNCIMB861666 were obtained from our laboratory. *E. coli* ATCC8739, *Staphylococcus aureus* ATCC29213, *Salmonella* Enteritidis ATCC14128, *S. aureus* ATCC6538, and *L. monocytogenes* ATCC 19115 were obtained from the Guangdong Culture Collection Center. Except for nisin-sensitive indicator strain *M. luteus* A1CNCIMB861666 and leucocin C-sensitive indicator strain *L. monocytogenes* WSLC 1018, all these strains are common foodborne pathogens and used as indicator in this study. These strains were cultured in LB broth (Hope Bio, Qingdao, China), except for *L. monocytogenes*, which was cultured in TSB-YE broth (Hope Bio, Qingdao, China).

Plasmids used in this study are listed in **Table 1**. pLEB124 was used to construct the expression vector. pNZ5319 was used to express *lecCI* (Leucocin C and its immunity gene) in the chromosome of N8 through Cre–*loxP* gene recombination system.

**Table 1:** Plasmids used in this study.

Plasmids	Relevant properties	Reference
pLEB690	*L. lactis* secretion vector harboring lactococcal promoters P_45_ and P*_nisZ_*, and signal sequence *SSusp45*	Li et al., 2011
pLEB729	Leucocin C and the immunity gene *lecCI* fused to *SSusp45* in pLEB690	Wan et al., 2013
pLEB124	*L. lactis* secretion vector harboring lactococcal promoter P_45_	Qiao et al., 1995
pLEB124-*lecCI*	fragment (P*_nisZ_*+*SSusp45*+*lecCI*) obtained from pLEB729 cloned into pLEB124	This study
pNZ5319	vector for gene replacements in Gram-positive bacteria	Lambert et al., 2007
pNZ5319-up-down	Upstream and downstream sequences of the *N8GL37-38* genes cloned into pNZ5319	This study
pNZ5319-up-down-*lecCI*	Fragment (P_45_+P*_nisZ_*+*SSusp45*+*lecCI*) obtained from pLEB124-*lecCI* replaced the *cat* gene in pNZ5319-up-down	This study

### Construction of Leucocin C Expression Strains

The plasmid pLEB729 (Wan et al., 2013) was digested with *Hin*dIII and *Bam*HI (Takara, Dalian, China) to obtain the expression fragment P*_nisZ_*+*SSusp45*+*lecCI*. The fragment was purified with DNA gel extraction kit (Solarbio, Beijing, China) and cloned into *Hin*dIII-*Bam*HI digested pLEB124 to produce pLEB124-*lecCI*. pLEB124-*lecCI* was then electroporated into *L. lactis* NZ9000 and *L. lactis* N8 to obtain NZ9000-p-lecCI and N8-p-lecCI (plasmid expression method) expression strains, respectively.

The non-necessary genes *N8GL37-38* were replaced with *lecCI* to stably express *lecCI* in the genome of N8 without antibiotic pressure. In brief, 633-bp upstream and 528-bp downstream sequences of the *N8GL37-38* genes were amplified with the N8 genome as template by using LA Taq (Takara, Dalian, China) and cloned into the *Xho*I-*Swa*I and *Sac*I–*Bgl*II restriction sites of pNZ5319, respectively, to obtain the pNZ5319–up-down vector. P_45_+P*_nisZ_*+*SSusp45*+*lecCI* was amplified with the pLEB124-*lecCI* as template by PCR using Phusion High-Fidelity DNA polymerase (Thermo Fisher, China) with primer pairs LecC F/R. PCRs were performed using the primers listed in **Table 2**. The PCR products and pNZ5319-up-down were digested with *Bgl*II and *Xho*I and then cloned into the *Bgl*II-*Xho*I sites of pNZ5319-up-down to replace the *cat* gene and obtain the recombination vector pNZ5319-up-down-*lecCI*, which was introduced into N8. The genomes of transformants and N8 were used as templates to amplify the target genes with the primers Ery-F/R, LecCI-F/R, and Out-F/R to identify the single or double cross-over recombinants. The PCR products were analyzed on 1% agarose gels. The potential nisin and leucocin C co-expression strain N8-r-lecCI (homologous recombination expression method) was identified.

**Table 2:** Primers used in this study.

Primer	Sequence (5′–3′)	Annealing temperature (°C)
**For the construction of leucocinC expression vector**		
**(pNZ5319-up-down-*lecCI*)**		
UP-F	CCGCTCGAG AGATGAGGGTAATCTGAAGGA	52
UP-R	CCCATTTAAAT ACCTTATCTTAAATGCCTATCTT	
Down-F	CCCGAGCTC GAAAGGGGGATTAGTGATCA	54
Down-R	GAAGATCT ACTCGCTACCGCCTGAAA	
LecCI-F	CGTTAGGGGCTTGAACAAG	56
LecCI-R	CTAATATCCATAACGAATACTAGAT	
**For the identification of leucocinC expression strain (N8-r-lecCI)**		
Ery-F	CGATACCGTTTACGAAATTGG	52
Ery-R	CTTGCTCATAAGTAACGGTAC	
Out-F	ACGAACAAAACAGTTGCG	52.5
Out-R	TTCTCTGCCATACTGGCTC	

### Growth Profile of Wild and Constructed Strains

N8, N8-p-lecCI, and N8-r-lecCI were cultured to OD_600_ of 0.8 in the SGM17 broth and diluted to an optical density of OD_600_ of 0.2. Afterward, 200 μl of the cultures were inoculated into 20 mL of SGM17 medium in a 50 mL triangular flask. Growth profiles were monitored by measuring OD_600_ for 12 h at 30°C. All tests were performed in triplicate.

### Anti-bacterial Activity

Agar diffusion assay was performed in triplicate to detect leucocin C and nisin activities of the culture supernatants of N8, N8-p-lecCI, N8-r-lecCI, and NZ9000-p-lecCI. In brief, 20 mL of soft agar and 80 μl of the corresponding indicator strain (5 × 10^8^ CFU/mL) were mixed and poured onto a plate with Oxford cups. The holes of the dried agar plates were added with 150 μl of the pasteurized supernatant (75°C, 10 min) from the overnight cultures of *L. lactis* strains. The plates were incubated overnight at corresponding temperature.

### SDS-PAGE and Identification of the Leucocin C Activity Band

Total protein was obtained from 100 mL of the cell-free overnight culture supernatants of NZ9000 (blank control) and NZ9000-p-lecCI (leucocin C) and precipitated by 50% ammonium sulfate at 4°C. The precipitate was centrifuged at 14,000 × *g* and 4°C for 30 min and released in 1 mL of sterile water. The concentrated supernatant samples were analyzed by tricine-SDS-PAGE (Schägger and von Jagow, 1987). Gels were prepared using 16% acrylamide resolving gel and 1 cm of 4% acrylamide spacer gel. After electrophoresis (Bio-Rad), the gel was divided vertically into two parts; the first part was stained with Coomassie brilliant blue G-250 (Takara, Dalian, China), and the second part was used for testing antimicrobial activity according to the method described earlier (Bhunia et al., 1987). The gel was washed for 30 min by sterile water and placed in a plate. The plate was poured with soft agar containing *L. monocytogenes* ATCC19115 and incubated at 37°C overnight.

### Comparison of Genetic Stability

The expression strains N8-p-lecCI (plasmid expression method) and N8-r-lecCI (homologous recombination expression method) were inoculated into 1.5 mL tube containing SMG17 broth (non-selective conditions). The strains were cultivated at 30°C for 8 h, transferred into a second tube, and cultivated under the same conditions. Serial transferring was carried out 70 times, and each transfer was referred to as 10 generation times (results not shown). A single colony was harvested to inspect the stability of the strain by calculating the percentage of leucocin C expressing cells in the population.

### Determination of the Minimum Inhibitory Concentration

The minimum inhibitory concentrations (MICs) of EDTA and N8-r-lecCI supernatant against *L. monocytogenes* WSLC1018, *L. monocytogenes* ATCC 19115, *Staphylococcus aureus* ATCC6538, *Staphylococcus aureus* ATCC29213, *E. coli* ATCC25922, *E. coli* O157:H7, *Salmonella* Enteritidis ATCC 14128 and *M. luteus* A1CNCIMB 861666 were determined using a broth micro-dilution assay (Clinical and Laboratory Standards Institute [CLSI], 2014). These strains were adjusted to a final concentration of 10^5^ CFU/mL in the corresponding broth, and 100 μl of the prepared suspension was added to the twofold serial dilutions of antibacterial agents (100 μl). After 24 h of incubation at 37°C, MIC was defined as the lowest antimicrobial concentration that did not result in visible growth. All tests were performed in triplicate.

### Checkerboard Assay

Checkerboard method (Martinez-Irujo et al., 1996) is usually used to evaluate the interaction of different drugs. In this study, each combination (A × B) of compound A (50 μl of EDTA) and B (50 μl of N8-r-lecCI supernatant) was diluted by twofold along the ordinate and abscissa axes in a 96-well microtiter plate. The final concentrations of the compounds ranged from 1/32–2 times the MIC for EDTA and 1/64–2 times the MIC for the N8-r-lecCI supernatant. The pathogenic bacteria were adjusted to the final concentration of 10^5^ CFU/mL in the corresponding broth (100 μl) and added to the combinations of the compounds. The plate was incubated at 37°C for 24 h. All tests were performed in triplicate.

### Time-Kill Assay in Pasteurized Milk

*Listeria monocytogenes*, as a common food contaminating bacterium poses significant threat to public health (Jemmi and Stephan, 2006). According to preliminary checkerboard assays, the positive interaction between EDTA and the N8-r-lecCI supernatant against *L. monocytogenes* ATCC 19115 was chosen for time–kill assay in pasteurized milk. The growth curve-based time–kill assay was determined according to a previous method with minor modification (Garcia et al., 2010; Shi et al., 2017). The experimental tubes containing pasteurized milk initially inoculated with 10^5^ CFU/mL pathogens were added with either single antibacterial agent or the combination of antibacterial agents. The tube with pathogen was used as control. The samples were cultured at 4°C (refrigeration temperature) and 25°C (room temperature) for 24 h. Bacterial counts were determined by spreading appropriate dilutions on TSB-YE agar every 4 h. The plates were incubated at 37°C overnight, and bacterial colonies were counted. In these experiments, the concentration of EDTA was consistent with 1/2 of the MIC, and the concentration of the N8-r-lecCI supernatant was consistent with the MIC. All tests and measurements were performed in triplicate.

### Scanning Electron Microscopy (SEM)

Logarithmic-phase bacteria (*L. monocytogenes* ATCC 19115) were collected at 4000 g for 5 min and washed twice with PBS (pH 7.4). The cells were suspended in PBS (OD_600_ = 0.2) and treated with EDTA (0.125 mg/mL)/N8-r-lecCI supernatant (MIC) or their combination [EDTA (0.125 mg/mL) and N8-r-lecCI supernatant (MIC)] for 1 h. The bacterial cells without antimicrobial treatment were similarly processed and used as controls. After incubation, the cells were washed with PBS and fixed overnight at 4°C with 2.5% glutaraldehyde. The cells were washed in the same buffer and dehydrated using gradient ethanol concentrations ranging from 50 to 100%, with each gradient for about 15 min. Ethanol was replaced with tertiary butyl alcohol. The samples were drop on the plate and prepared for SEM (QUANTA 200) observation.

## Results

### Identification of Activity and Protein Band of Leucocin C

**FIGURE 1:**
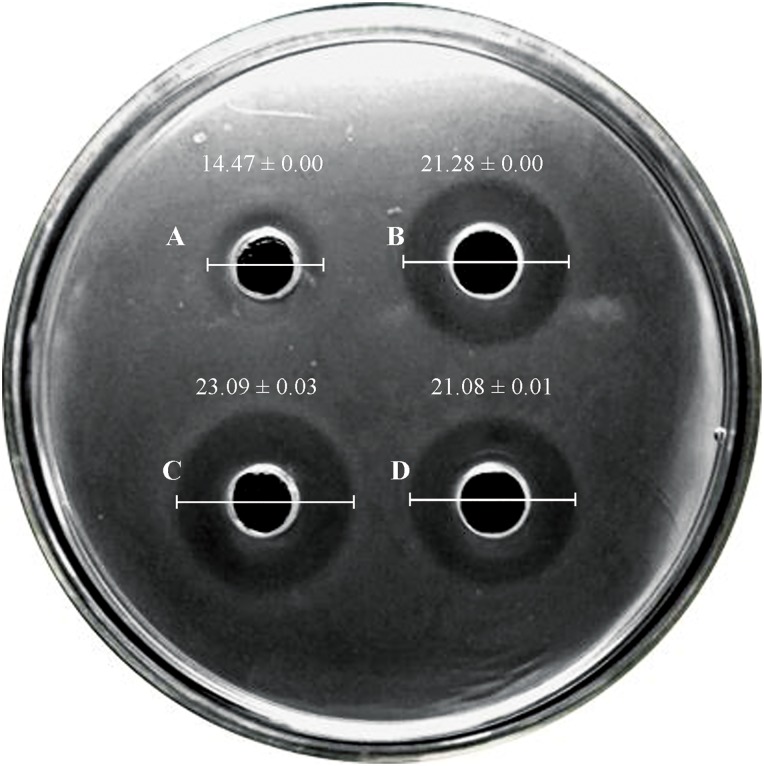
Results of agar-diffusion experiment with leucocin C-producing *L*. *lactis* and the wild type N8 (indicator strain: *L. monocytogenes* WSLC 1018). Three independent experiments were conducted, and the image represents the results of one of the experiments (unit: mm). **(A)** *L. lactis* N8; **(B)** *L. lactis* N8-p-lecCI; **(C)** *L. lactis* N8-r-lecCI; and **(D)** *L. lactis* NZ9000-p-lecCI. The difference between two groups was compared by *t*-test, the diameter of inhibitions zone of N8-p-lecCI, N8-r-lecCI and NZ9000-p-lecCI were significantly different (*P* < 0.05) compared with N8.

**FIGURE 2:**
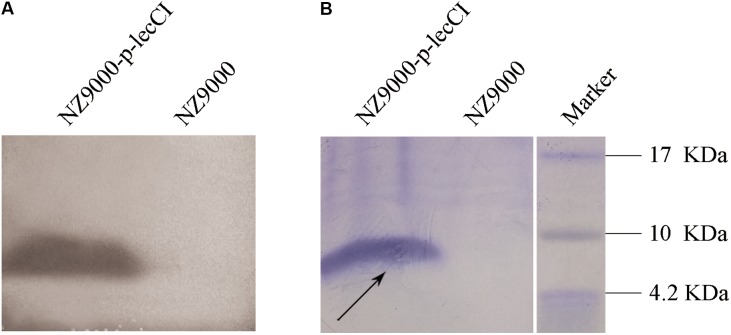
SDS-PAGE of supernatant concentrations from overnight cultures of *L. lactis* NZ9000 (control) and leucocin C-producing strain *L. lactis* NZ9000-p-lecCI. **(A)** The gel was covered with soft agar containing *L. monocytogenes* WSLC1018. **(B)** Coomassie blue G-250-stained gel. A putative leucocin C band (black arrow) in **(B)** and the corresponding inhibition zone in **(A)** appeared between 4.2 and 10 kDa. The actual size of leucocin C is about 4.6 kDa.

Bacteriocin production was determined by agar diffusion assay on a plate. Leucocin C was effectively secreted by recombinant *L. lactis* strains and shown as inhibition zones on *L. monocytogenes* indicator plates (**Figure 1**). All constructed strains showed excellent anti-listeriosis activity compared with the wild strain N8 (slight inhibition). The inhibitory effect of N8-r-lecCI was more distinct than the plasmid expression strains N8-p-lecCI and NZ9000-p-lecCI, indicating the superior antibacterial activity of N8-r-lecCI. The proteins from the culture supernatants were concentrated and analyzed with SDS-PAGE to confirm whether or not leucocin C inhibits the growth of *L. monocytogenes*. Results of Coomassie brilliant Blue G-250 staining indicated the migration of the putative leucocin C band between 4.2 and 10 kDa (**Figure 2B**). An inhibition zone was found around the potential leucocin C band in the second part of the gel which was used for testing antimicrobial activity against *L. monocytogenes*, whereas no antimicrobial activity was detected in the remaining parts of the gel (**Figure 2A**). This finding suggested that the protein band in SDS-PAGE is leucocin C. All these results indicated that the constructed strains can successfully secrete leucocin C.

### Growth Profiles of Wild and Constructed Strains

**FIGURE 3:**
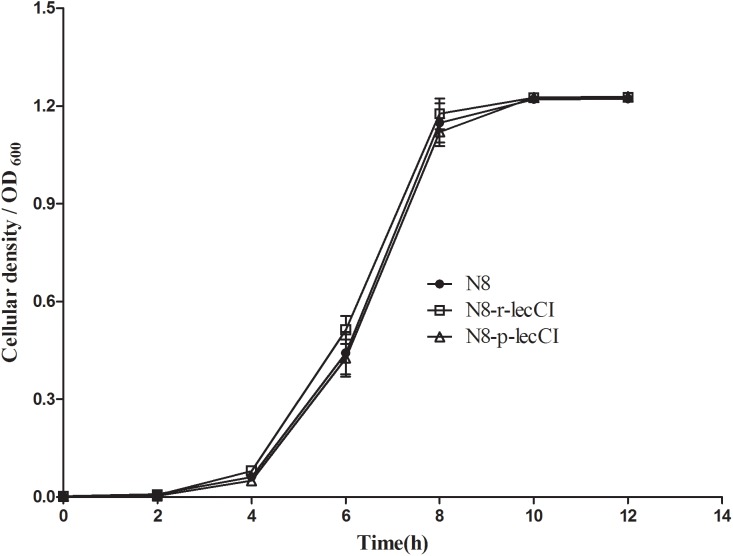
Growth profiles of N8, N8-p-lecCI and N8-r-lecCI in SMG17 (non-selective pressure). The cellular density of the cultures was monitored once every 2 h by measuring OD600 for 12 h. All growth curve experiments were carried out in triplicate, and each point represents the average of separate experiments with standard deviation indicated by vertical bars. The difference between two groups was compared by *t*-test, the growth profiles of different strains exhibited no significant difference.

The growth curves of N8, N8-p-lecCI, and N8-r-lecCI in SMG17 (no-selective pressure) are shown in **Figure 3**. The growth profiles were similar among the wild-type strain N8, N8-p-lecCI, and N8-r-lecCI. Hence, the growth of N8 was not affected by the expression of leucocin C.

### Determination of Genetic Stability

The genetic stability of different *lecCI* expression methods was compared. The genetic stability of N8-r-lecCI and N8-p-lecCI was tested by continuously reculturing in SGM17 broth without selective pressure for 500 and 700 generation times. At the 500th generation time, the percentage of leucocin C expression cells of population of N8-p-lecCI reduced to 99.33 ± 0.47% and this figure dropped to 98.33 ± 0.47% after 700 generations. By contrast, N8-r-lecCI maintained excellent genetic stability (the percentage of leucocin C expression cells is 100%) after 700 generation times. Hence, homologous recombination (*lecC*-R expression method) was superior to *lecC*-P expression method because the plasmid of N8-p-lecCI can be lost in the absence of antibiotic pressure.

### Determination of Antimicrobial Spectrum and Antimicrobial Activity

**FIGURE 4:**
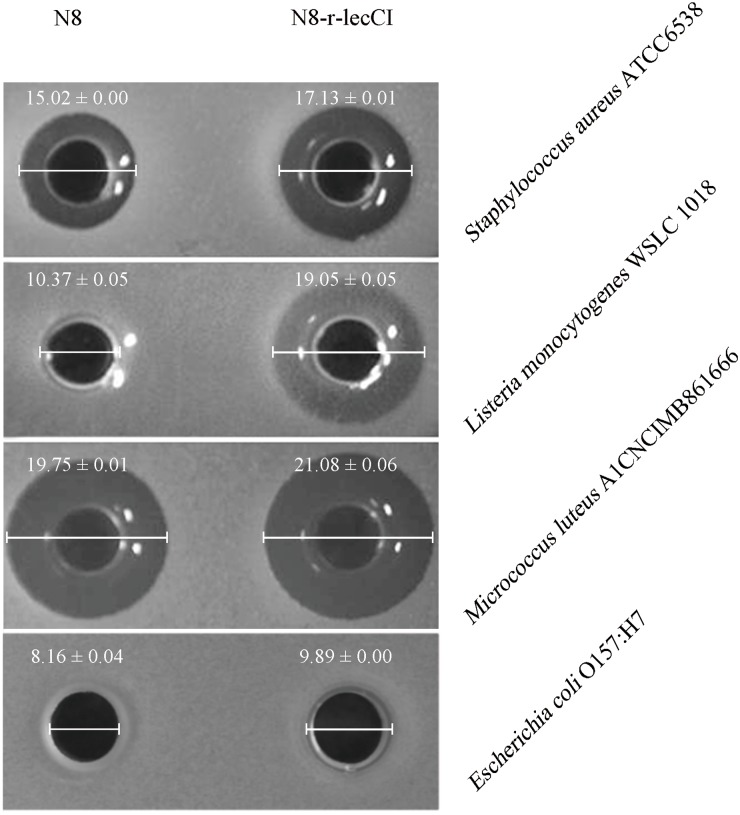
Anti-bacterial assays comparing the antimicrobial activities of co-expression strain *L. lactis* N8-r-lecCI and wild-type N8 against *S. aureus* ATCC6538, *L. monocytogenes* WSLC 1018, *M. luteus* A1CNCIMB861666, and *Escherichia coli* O157:H7. The holes of the plates were added with 150 μl of the pasteurized supernatant (75°C, 10 min) obtained from overnight cultures of *L. lactis* strains. The diameter of the inhibition zones represents differences in antimicrobial activity. The difference between two groups was compared by *t*-test, the antimicrobial activity of N8-r-lecCI against four indicator strains was significantly different (*P* < 0.05) compared with N8 (unit: mm).

To verify the advantages of the co-expression strain N8-r-lecCI in terms of scope and activity of bacteriostasis, we compared it with the wild-type strain N8. The results of antimicrobial spectrum analysis are shown in **Table 3**. The pasteurized supernatant (75°C for 10 min) from N8-r-lecCI inhibited the growth of the eight strains tested, and the wild-type strain N8 inhibited only four strains. The antimicrobial spectrum of the recombinant strain N8-r-lecCI evidently increased. As shown in **Figure 4**, the co-expression strain N8-r-lecCI was superior to the parental strain N8 in terms of bacteriostatic activity against *S. aureus* ATCC6538, *L. monocytogenes* WSLC 1018, *M. luteus* A1CNCIMB861666, and *E. coli* O157:H7.

**Table 3:** Antimicrobial spectrum of N8 and N8-r-lecCI.

Indicative bacteria	Inhibitory effect	
	N8	N8-r-lecCI
*S. aureus* ATCC 6538	+++	++++
*S. aureus* ATCC 29213	+++	++++
*L. monocytogenes* WSLC 1018	-	+++
*L. monocytogenes* ATCC 19115	+	+++
*E. coli* ATCC 8739	-	+
*E. coli* O157:H7	-	+
*S.* Enteritidis ATCC 14128	-	+
*M. luteus* A1CNCIMB 861666	+++	+++

*(-), absence of inhibitory effect with twofold dilution of supernatant; (+), presence of inhibitory effect with twofold dilution of supernatant; (++), presence of inhibitory effect with fourfold dilution of supernatant; (+++), presence of inhibitory effect with eightfold dilution of supernatant; (++++), presence of inhibitory effect with 32-fold (greater or equal to) dilution of supernatant*.

### Effects of N8-r-lecCI Supernatant Alone or in Combination With EDTA

The activities of the N8-r-lecCI supernatant and EDTA against three common foodborne pathogens alone or in combination are summarized in **Table 4**. The N8-r-lecCI supernatant and EDTA showed different antimicrobial activities against the tested strains based on the calculated MICs. The MIC of the N8-r-lecCI supernatant decreased by 75% in the presence of 0.0625 mg/mL EDTA against *L. monocytogenes* ATCC 19155 and decreased by 75% in the presence of 0.0312 mg/mL EDTA against *S. aureus* ATCC 6538. The combination of EDTA and N8-r-lecCI supernatant exhibited bactericidal effect on *E. coli* O157:H7, and the MIC of the N8-r-lecCI supernatant decreased by 87.5% in the presence of 0.625 mg/mL EDTA. EDTA and the N8-r-lecCI supernatant showed weak or even no antibacterial activities when used alone at this low concentration.

**Table 4:** Minimum inhibitory concentrations (MICs) of *L. lactis* N8-r-lecCI supernatants alone and in combination with EDTA against common foodborne strains.

Strains	MIC (range) of compounds			
	Alone		Combination	
	EDTA (mg/mL)	Supernatant	EDTA (mg/mL)	Supernatant
*L. monocytogenes* ATCC 19115	0.25	1/8×	0.0625	1/32×
*S. aureus* ATCC 6538	0.0625	1/64×	0.03125	1/256×
*E. coli* O157:H7	2.5	1/2×	0.625	1/16×

*(×), multiple*.

### Time-Kill Assay in Pasteurized Milk

**FIGURE 5:**
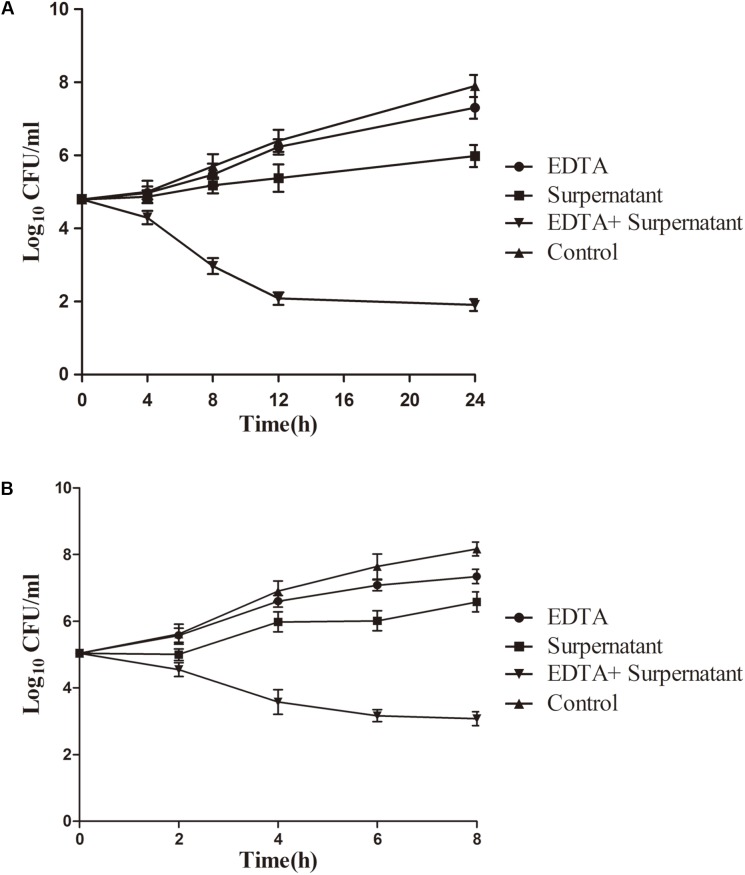
Time-kill curves for EDTA and N8-*lecCI* supernatant alone or in combination against *L. monocytogenes* ATCC 19155 in pasteurized milk at **(A)** refrigeration temperature (4°C) and **(B)** room temperature (25°C). The strains were treated with EDTA (0.125 mg/mL), N8-r-lecCI supernatant (concentration of the MIC), and EDTA (0.125 mg/mL) + N8-r-lecCI supernatant (concentration of the MIC) at a starting inoculum concentration of 10^5^ CFU/mL. All time–kill curves were constructed in triplicate, and each point represents the average of separate experiments with standard deviation indicated by vertical bars. The difference between two groups was compared by *t*-test, the antimicrobial effect of EDTA + N8-r-lecCI supernatant was significantly different (*P* < 0.05) compared with EDTA, N8-r-lecCI supernatant and control.

The increased antibacterial activity of the combination of EDTA and the N8-r-lecCI supernatant was further investigated in pasteurized milk to evaluate the practical application of this method. Considering the growth of *L. monocytogenes* at low temperature, we performed the assays at 4 and 25°C. As shown in **Figure 5**, bacterial growth was more effectively inhibited in the sample treated with the mixture of EDTA and N8-r-lecCI supernatant at both temperatures compared with that in the sample treated with a single antibacterial substance. The combination of EDTA and the N8-r-lecCI supernatant reduced the viable bacterial count by more than 2 log_10_ CFU/mL at both temperatures, indicating a positive interaction (Knezevic et al., 2013). The effect of the combination in pasteurized milk corresponded to the results presented in checkerboard assay. The results revealed that the combination of EDTA and N8-r-lecCI supernatant exerts a strong bactericidal effect in pasteurized milk against *L. monocytogenes*.

### Effect of Antimicrobials on Bacterial Morphology

**FIGURE 6:**
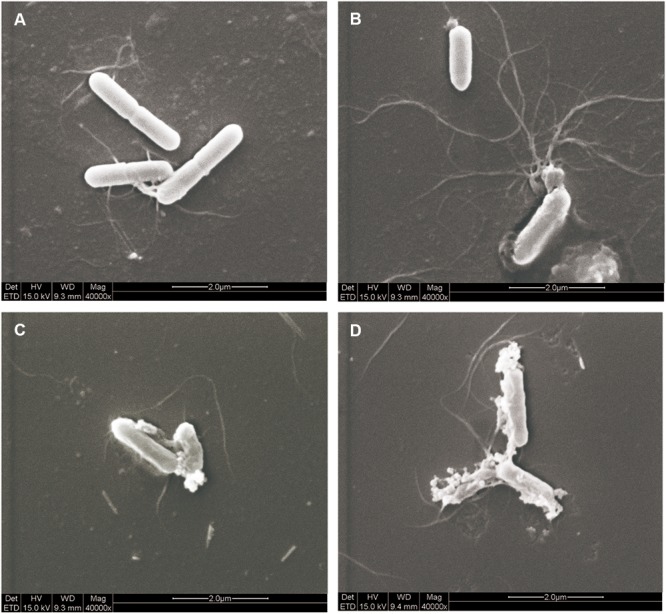
Scanning electron microscopy (SEM) observation of *L. monocytogenes* ATCC 19155 treated with EDTA and N8-r-lecCI supernatant alone and in combination. **(A)** Control cells (no treatment), **(B)** cells treated with EDTA (0.125 mg/mL), **(C)** cells treated with the N8-r-lecCI supernatant (concentration of the MIC), and **(D)** cells treated with EDTA and the N8-r-lecCI supernatant.

Morphological changes in *L. monocytogenes* cells were observed through SEM analysis to intuitively elucidate the antibacterial effect of EDTA and the N8-r-lecCI supernatant alone or in combination. As shown in **Figure 6**, cell damage was observed considering the evident changes in the cell morphology. The control cells maintained a relatively intact cell morphology with a smooth surface (**Figure 6A**), and the cells treated with EDTA at the 1/2 MIC concentration showed slight damage (**Figure 6B**). Moreover, the cells treated with the N8-r-lecCI supernatant at the MIC concentration showed relatively more severe damage than those treated with EDTA alone (**Figure 6C**). The significant cell damage was observed in the sample treated with EDTA + N8-r-lecCI supernatant. Most of the outermost layer of the bacterial cells disappeared, and the cell inclusions leaked, leading to the death of bacteria. These results revealed that the inhibition of bacterial growth could be due to the membrane disruption of cells, that is, a stronger antibacterial activity causes more severe cell damage. This assay confirmed that the combination of EDTA and N8-r-lecCI exhibited strong antibacterial activity.

## Discussion

The food-grade strain *L. lactis* N8 was used as the original strain because of its potential as nisin-leucocin C co-expression carrier. The results of agar diffusion assay and SDS-PAGE verified that the constructed strains can successfully secrete leucocin C and nisin, and the secreted leucocin C exhibited distinct inhibitory effect against *Listeria monocytogenes*. It is also worth noting that the expression of leucocin C did not influence the growth of strains.

Accumulating evidences indicated that serial passage of plasmid-borne strains expressing a protein often result in genetic instability (O’Kennedy et al., 2000), which is an important factor that should be considered when developing recombinant strains for commercial application in industrial fermentation. Therefore, the genetic stability of different *lecCI* expression methods was compared. Homologous recombination method (N8-r-lecCI) was superior to plasmid expression method (N8-p-lecCI) because the plasmid of N8-p-lecCI can be lost in the absence of antibiotic pressure. In addition, the relative advantage of N8-r-lecCI compared with N8-p-lecCI is that non-food source gene was introduced into the expression host. This result verified that the recombinant strain N8-r-lecCI has excellent genetic stability and can stably coexpress nisin–leucocin C, which means it is more suitable for food fermentation and application.

The co-production strain N8-r-lecCI exhibits broad activity spectrum and highly efficient bacteriostatic ability against Gram-positive foodborne pathogens. Several studies have demonstrated that nisin show additive or synergistic effects when used in combination with other antimicrobial agents (Branen and Davidson, 2004; Field et al., 2016; Shi et al., 2017). However, to the best of our knowledge, no information regarding the antibacterial activity of nisin co-expression with leucocin C or in combination with leucocin C was available in literature. Our study first demonstrated that the co-expression of these two bacteriocins which have different modes of action results in highly efficient antimicrobial activity against *Listeria monocytogenes* and *Staphylococcus aureus*. It is a novel combination that is worthy of further investigation.

The food grade chelators EDTA and the N8-r-lecCI supernatant were combined to further enhance the antimicrobial activity of the N8-r-lecCI supernatant. Previous studies have demonstrated that EDTA could be used in the food industry due to its active antibacterial activity and its safety (Heimbach et al., 2000). Our study showed the synergistic antibacterial activity of N8-r-lecCI supernatant and EDTA against *L. monocytogenes* WSLC1018 and *E. coli* ATCC25922, and enhanced activity against *S. aureus* ATCC 6538. Previous reports described that the combination of nisin and EDTA can enhance antimicrobial ability against Gram-negative bacteria and *L. monocytogenes* (Zhang and Mustapha, 1999; Mastromatteo et al., 2010; Norhana et al., 2012). Several studies have explained the reason regarding the antibacterial efficiency of the combination of nisin and EDTA (Branen and Davidson, 2004). EDTA could release up to 50% of the lipopolysaccharide (LPS) to affect the outer membrane permeability (Leive, 1965), thereby increasing the susceptibility of cells to nisin. Hence, we hypothesized that EDTA could contribute to efficient binding of leucocin C to mannose phosphotransferase permease, which serves as target in sensitive cells (Kjos et al., 2009; Wan et al., 2013). This assumption must be further investigated. In the present study, the results revealed the positive interaction among leucocin C, nisin, and EDTA against some foodborne strains. In addition, the organic acids of the N8-r-lecCI fermentation supernatant helped to suppress the pathogens. Organic acids are widely used for food preservation (Young and Foegeding, 1993). The antimicrobial activity of nisin or other bacteriocins against *L. monocytogenes* F6854 can be enhanced in the presence of organic acids (Smith et al., 2016). Overall, the antibacterial activity of *L. lactis* N8-r-lecCI supernatant can be further enhanced in the presence of the low concentration of EDTA.

The practical antibacterial effect of this combination was verified in a food model through time-kill assay. The effect of the combination in pasteurized milk was consistent with the result presented in checkerboard assay, which shows excellent antimicrobial activity in pasteurized milk preservation. The current standards of pasteurization appear to be adequate for public health assurance of milk safety (Holsinger et al., 1997). However, outbreaks associated with pasteurized milk continue to occur (De Buyser et al., 2001; Centers for Disease Control and Prevention, 2008). The combination of bacteriocins has excellent antimicrobial potential in pasteurized milk preservation.

## Conclusion

*Lactococcus lactis* N8-r-lecCI co-expressing nisin and leucocin C is characterized by high genetic stability and antibacterial efficiency, and exhibits high potential in food preservation and processing.

## Author Contributions

DZ, YF, and MQ designed the experiments. YF, XW, and WQ performed all the major experiments. DM, FL, and MQ analyzed the experimental results. DM and PS provided the additional experimental assistance. YF and DZ wrote the manuscript. OK and MQ helped to revise the manuscript. MQ and HX provided the laboratory equipment and place.

## Conflict of Interest Statement

The authors declare that the research was conducted in the absence of any commercial or financial relationships that could be construed as a potential conflict of interest.

## Abbreviations

EDTA: ethylenediaminetetraacetic acid
*lecCI*: bacteriocin leucocin C and its immunity gene.
